# Diagnostic value of circN4BP2L2 in type I and type II epithelial ovarian cancer

**DOI:** 10.1186/s12885-022-10138-w

**Published:** 2022-11-24

**Authors:** Li Ning, Jinghe Lang, Bo Long, Lingying Wu

**Affiliations:** 1 Department of gynecologic oncology, National Clinical Research Center for Cancer/Cancer Hospital, National Cancer Center, Chinese Academy of Medical Sciences and Peking Union Medical College, 100021 Beijing, China; 2 Peking Union Medical College Hospital, Chinese Academy of Medical Sciences and Peking Union Medical College, 100730 Beijing, China

**Keywords:** Epithelial ovarian cancer, Circular RNA, Human, Biological markers, Diagnosis

## Abstract

**Background:**

CircN4BP2L2 was previously identified to be significantly decreased in epithelial ovarian cancer (EOC) and was associated with disease progression. The aim of this study was to evaluate the diagnostic value of plasma circN4BP2L2 using the unifying model of type I and type II EOC.

**Methods:**

A total of 540 plasma samples were obtained from 180 EOC patients, 180 benign ovarian cyst patients, and 180 healthy volunteers. CircN4BP2L2 was assessed using reverse transcription-quantitative polymerase chain reaction (RT-qPCR). Cancer antigen 125 (CA125) and human epididymis protein 4 (HE4) were assessed using enzyme-linked immunosorbent assay (ELISA). Receiver operating curve (ROC), the area under the curve (AUC), sensitivity and specificity were estimated.

**Results:**

Low level of circN4BP2L2 was associated with advanced tumor stage (*p* < 0.01) in type I EOC. Decreased circN4BP2L2 was associated with lymph node metastasis (LNM) (*p* = 0.04) in type II EOC. The expression level of circN4BP2L2 in type I was similar to that in type II. CircN4BP2L2 could significantly separate type I or type II from benign or normal cohort (*p* < 0.01). Early-stage type I or type II EOC vs. benign or normal cohort could also be distinguished by circN4BP2L2 (*p* < 0.01).

**Conclusion:**

CircN4BP2L2 might serve as a promising diagnostic biomarker for both type I and type II EOC. The diagnostic safety for circN4BP2L2 in early-stage type I or type II EOC is also acceptable. Further large-scale well-designed studies are warranted to investigate whether circN4BP2L2 is specific for all histologic subgroups.

**Supplementary Information:**

The online version contains supplementary material available at 10.1186/s12885-022-10138-w.

## Background

Ovarian cancer (OC) is the most lethal malignancy in female reproductive system [1]. The number of new cases and deaths of OC worldwide was estimated at 295,000 and 187,000 annually [2]. Despite recent progress in surgical skills and targeted drugs, most advanced patients still develop tumor recurrence in three years after OC diagnosis, and relapsed diseases were usually incurable [3]. The 5-year survival rate of OC patients is merely 40% [3]. Epithelial ovarian cancer (EOC) is the most common subtype, accounting for approximately 95% of all OC cases [4]. Due to lacking of specific early symptoms and reliable screening tests, over 70% of EOC patients are diagnosed at advanced stages [5]. Owing to the great heterogeneity in biological and molecular status, EOC is actually consisting of many different tumors [6]. Based on morphologic features, the four major histological subtypes of EOC are, serous, mucinous, endometrioid and clear cell [6]. EOC has been thought to arise from epithelial cells lining the surface of the ovary [7]. Recent findings suggest that EOC may also arise from the epithelium of fallopian tube and endometriosis [8].

Based on distinct morphological features and molecular genetics, EOC is further divided into type I and type II tumors [9]. Type I tumors include low-grade (G1) serous, low-grade (G1) endometrioid, all clear cell, mucinous and Brenner carcinomas [10]. These tumors are generally indolent, more often diagnosed at stage I (tumor confined to the ovary), harbor specific gene mutations such as KRAS, CTNNB1 and ARID1A, have relatively stable genome and rarely have TP53 mutation [9–11]. Type II tumors include high-grade (G2–G3) serous, high-grade (G2–G3) endometrioid, all undifferentiated carcinoma and malignant mixed mesodermal tumors (carcinosarcomas) [10]. These tumors are more aggressive, more often present at advanced stage, harbor highly unstable genome, have a high frequency of TP53 mutation had have molecular alterations that causing BRCA dysfunction via gene mutation or promoter methylation [9, 10, 12].

Cancer antigen 125 (CA125) is the first biomarker for OC approved by the US Food and Drug Administration (FDA). Nevertheless, CA125 is limited by its relatively low sensitivity and low specificity [13]. It has been reported that CA125 was elevated in only 50–60% of early-stage tumors [14]. Human epididymal protein 4 (HE4) is the second FDA-approved OC biomarker [15]. Although HE4 has a relatively high specificity since it does not increase in benign gynecological diseases, it is also limited by its relatively low sensitivity (approximately 70%) [16].

Circular RNAs (circRNAs) are a novel subtype of non-coding RNAs [17]. Their high stability, abundance, conservation and tissue-specificity make them promising biomarkers for disease detection [18]. A body of clinical trials have been performed using blood samples to evaluate the diagnostic value of circRNAs in various diseases, including cancer. Ouyang et al. [19] reported that plasma circ_002453 was significantly increased in patients with lupus nephritis and might serve as a potential biomarker for disease diagnosis. Huang et al. [20] reported that plasma circ_0001953 and circ_0009024 were remarkably elevated in patients with active tuberculosis and might represent novel diagnostic biomarkers. In Zhu et al.’s study [21], circ_0027089 exhibited high abundance in plasma and could distinguish hepatitis B virus-related hepatocellular carcinoma from cirrhosis or healthy controls. In Liu et al.’s study [22], a two-circular RNA signature was identified as a promising noninvasive diagnostic biomarker for lung adenocarcinoma.

Recent studies showed that circRNAs participated in EOC tumorigenesis and progression by regulating various processes, including cell proliferation, apoptosis, invasion and migration. Ding et al. [23] reported that circ_0072995 could promote cell proliferation and migration via modulating miR-147a/CKD6 axis in EOC. Gan et al. [24] reported that circMUC16 could promote autophagy of EOC via interaction with miR-199a and ATG13. Additionally, circRNAs are reported to serve as potential biomarkers for EOC diagnosis and prognosis. In Liu et al.’s study [25], circHIPK3 was upregulated and predicted a poor prognosis in EOC. In Hu et al.’s study [26], plasma circBNC2 represented a promising diagnostic biomarker for EOC.

In our previous experiment, we found that circN4BP2L2 was significantly downregulated in EOC by performing circRNA-sequencing analysis [27]. Besides, low level of circN4BP2L2 was predictive of disease progression [27]. Functional experiments revealed that downregulation of circN4BP2L2 could promote cell migration and invasion in EOC cell line [28]. Furthermore, plasma circN4BP2L2 could significantly distinguish EOC from benign ovarian cyst and healthy controls [28]. In this study, we aimed to further evaluate the diagnostic value of circN4BP2L2 by the unifying model of type I and type II EOC.

## Methods

### Study population

A total of 559 women were initially enrolled in this study. Nineteen malignancies were excluded because of non-EOC (n = 10, 6 granulosa cell tumors, 2 endodermal sinus tumors, and 2 dysgerminomas) and metastasis (n = 9). The eligible study population (n = 540) consisted of age and menopause-matched women with EOC (n = 180), benign ovarian cyst (n = 180), and healthy volunteers (n = 180). The study protocol was approved by the local ethics committee at the National Cancer Center/National Clinical Research Center for Cancer/Cancer Hospital and Peking Union Medical College Hospital of Chinese Academy of Medical Sciences (CAMS), and was conducted in accordance with the Declaration of Helsinki. Written informed consents for sample collection and article publication were obtained from all enrolled women.

After surgery, the tumors were independently examined by two experienced pathologists for the diagnosis of EOC, tumor histology, tumor grade and tumor stage (I–IV), according to the International Federation of Gynecology and Obstetrics (FIGO) standards. The exclusion criteria were: ovarian borderline tumors; patients with preoperative chemotherapy, radiotherapy or target therapy; and patients with other coexisting malignancies. The EOCs were further divided into type I and type II tumors.

### Sample collection

Patients were prospectively and consecutively enrolled when admitted for surgery for suspicious malignancies or benign ovarian cysts at the Department of Gynecologic Oncology in Cancer Hospital and Peking Union Medical College Hospital of CAMS, between December 2015 and April 2021. Blood samples were obtained on the surgery day. After centrifugation (3000 rotations per minute for 5 min), plasma samples were separated and stored at − 80 °C until use.

### RNA preparation and quality assessment

Total RNA was extracted from 200 µL of plasma samples using TRIzol reagent (Takara Bio, Nojihigashi, Kusatsu, Japan) according to the manufacturer’s instructions. RNA concentration was measured using NanoDrop 1000 spectrophotometers, and RNA was set at an OD A260/280 ratio between 1.8 and 2.1 and an OD A260/230 ratio > 1.8.

### RT-qPCR

RT-qPCR was conducted using PrimeScript™ RT reagent Kit with gDNA Eraser (Takara Bio, Nojihigashi, Kusatsu, Japan) and SYBR® Premix Ex Taq™ II (Tli RNaseHPlus) (Takara Bio, Nojihigashi, Kusatsu, Japan) according to the manufacturer’s instructions. GAPDH was used as an internal reference gene. The RT-qPCR protocol included a denaturation step (95 °C for 30 s) and 40 cycles of denaturation (95 °C for 5 s) and annealing (60 °C for 40 s). The relative expression level was calculated using the 2^−ΔΔCT^ method. The primer sequences were as follows: circN4BP2L2 (forward, 5′-CATGGTGTGTCTCGAAAGAAG-3′ and reverse, 5′-CTGTACCCATC TTGATGGTGA-3′) and GAPDH (forward, 5ʹ-AACGTGTCAGTGGTGGACCTG-3ʹ and reverse, 5ʹ-GAGACCACCTGGTGCTCAGTG-3ʹ).

### ELISA

ELISA analyses were conducted on coded plasma samples to measure the CA125 levels (Quantikine Human CA125 Immunoassay; R&D Systems, Minneapolis, USA) and HE4 concentrations (Quantikine Human HE4 Immunoassay; R&D Systems, Minneapolis, USA) according to the manufacturer’s instructions.

### Statistical analysis

SPSS 24.0 (SPSS Inc., Chicago, IL, USA) were used to conduct the statistical analyses. The statistical differences between two groups were compared by the Mann-Whitney test. The statistical differences among three or more groups were compared using the one-way ANOVA test. Youden index (specificity + sensitivity-1) was used to calculate the cut-off value of circN4BP2L2 (Supplementary Table S1). The cut-off value for CA125 < 35 U/mL and for HE4 < 55.86 pmol/L were adopted. Cases with marker levels above (CA125 and HE4) or below (circN4BP2L2) threshold levels were considered to be positive. The probabilities for each marker were predicted by constructing the receiver operating characteristic (ROC) curves. The area under the curve (AUC) value, sensitivity (sen) and specificity (spe) were calculated for individual markers. A value of *p* < 0.05 was considered to be statistically significant.

## Results

### Patient material

Of 540 women eligible for analysis, 180 had EOC, 180 had benign ovarian cyst, and 180 were healthy controls. The average age was 56 (range, 30–76) years old. The malignancies were further divided into type I (n = 70, 39%) and type II (n = 110, 61%) EOC based on histology and grade. Type I included low-grade serous (n = 16; 9%), low-grade endometrioid (n = 15; 8%), all clear cell (n = 23; 13%) and all mucinous (n = 14; 8%). Type II included high-grade serous (n = 88; 50%), high-grade endometrioid (n = 13; 7%), all undifferentiated (n = 7; 4%) and all malignant mixed mesodermal tumors (n = 2; 1%). Most patients were diagnosed at advanced stages (FIGO III + IV: 116/180, 65%). The benign cohort included endometriosis (n = 104, 58%), serous cystadenoma (n = 24, 13%), mucinous cystadenoma (n = 29, 16%), simple cystadenoma (n = 10, 6%) and mature teratoma (n = 13, 7%) (Table 1).

**Table 1:** The main clinicopathologic parameters of eligible women (n = 540)

	n (%)
Age, average, range ^a^	56 (30–76)
Epithelial ovarian cancer ^b^	
EOC type I	70/180 (39%)
EOC type II	110/180 (61%)
Histological subtype	
Serous carcinoma Low-grade High-grade	104/180 (59%) 16/180 (9%) 88/180 (50%)
Endometrioid carcinoma Low-grade High-grade	28/180 (15%) 15/180 (8%) 13/180 (7%)
Clear cell carcinoma	23/180 (13%)
Mucinous carcinoma Undifferentiated carcinoma Malignant mixed mesodermal tumor	14/180 (8%) 7/180 (4%) 2/180 (1%)
FIGO stage	
I	47/180 (26%)
II	17/180 (9%)
III	97/180 (54%)
IV	19/180 (11%)
Tumor grade	
High	54/180 (30%)
Moderate Poor Undifferentiated	35/180 (19%) 84/180 (47%) 7/180 (4%)
Benign ovarian cyst ^c^	
Endometriosis	104/180 (58%)
Serous cystadenoma	24/180 (13%)
Mucinous cystadenoma Simple cystadenoma	29/180 (16%) 10/180 (6%)
Mature teratoma	13/180 (7%)

Notes: ^a^ Average age of 540 age-matched eligible women. ^b^ The clinicopathologic parameters of patients with epithelial ovarian cancer (n = 180). ^c^ Histological subtype of patients with benign ovarian cyst (n = 180)

Abbreviations: n, number; EOC, epithelial ovarian cancer; FIGO, International Federation of Gynecology and Obstetrics

### CircN4BP2L2 level and clinicopathologic features of type I and type II EOC

The relationship between circN4BP2L2 level and clinicopathologic features of EOC was assessed in 180 patients (Table 2). The results showed that decreased circN4BP2L2 was associated with advanced FIGO stage (*p* < 0.01), worse tumor grade (*p* = 0.03) and LNM (*p* < 0.01) in EOC patients. CircN4BP2L2 level had no correlation to age or histology in EOC patients. A total of 70 patients with type I tumors were enrolled to evaluate the relationship between circN4BP2L2 and clinicopathological parameters. The data showed that low expression level of circN4BP2L2 was associated with advanced FIGO stage (*p* < 0.01), but not correlated to age, histology, tumor grade or LNM in type I EOC. A total of 110 type II EOC patients were included to investigate the association between circN4BP2L2 level and clinicopathological features. The results revealed that decreased circN4BP2L2 was associated with LNM (*p* = 0.04), but not related to age, histology or tumor stage in type II EOC.

**Table 2:** Correlation between plasma circN4BP2L2 expression level and clinicopathologic parameters of EOC (n = 180)

Parameter	EOC (n = 180)			Type I EOC (n = 70)			Type II EOC (n = 110)		
	n (%)	CircN4BP2L2, Mean ± SD	***p***	n (%)	CircN4BP2L2, Mean ± SD	***p***	n (%)	CircN4BP2L2, Mean ± SD	***p***
Age			0.52			0.70			0.47
≤ 50y	54 (30%)	27.48 ± 21.15		17 (24%)	30.24 ± 24.74		37 (34%)	26.21 ± 19.52	
> 50y	126 (70%)	26.70 ± 22.51		53 (76%)	31.04 ± 24.92		73 (66%)	23.54 ± 20.19	
Histology			0.75			0.11			0.76
Serous	106 (59%)	27.01 ± 22.88		18 (26%)	40.11 ± 30.81		88 (80%)	28.53 ± 22.19	
Others	74 (41%)	26.81 ± 20.98		52 (74%)	27.64 ± 21.64		22 (20%)	23.30 ± 19.22	
FIGO stage			< 0.01^*^			< 0.01^*^			0.44
I + II	64 (36%)	35.19 ± 25.19		40 (57%)	39.19 ± 26.29		24 (22%)	39.19 ± 26.29	
III + IV	116 (64%)	22.38 ± 18.73		30 (43%)	19.73 ± 17.27		86 (78%)	19.73 ± 17.27	
Grade			0.03^*^			0.15			NA
G1	54 (30%)	32.69 ± 25.61		54 (77%)	32.69 ± 25.61		0 (0%)	NA	
G2 + G3	126 (70%)	24.47 ± 19.96		16 (23%)	24.64 ± 20.88		110 (100%)	24.44 ± 19.92	
LNM ^a^			< 0.01^*^			0.70			0.04^*^
Yes	59 (40%)	19.71 ± 15.11		8 (14%)	19.27 ± 10.81		51 (57%)	19.78 ± 15.76	
No	89 (60%)	30.46 ± 23.00		50 (86%)	31.61 ± 24.55		39 (43%)	29.00 ± 21.07	

Notes: ^a^ The information for lymph node metastasis was only available in 148 EOC patients

Abbreviations: n, number; FIGO, International Federation of Gynecology and Obstetrics; G, grade; LNM, lymph node metastasis; SD, standard deviation; NA, not available

### Levels of circN4BP2L2, CA125 and HE4 in type I and type II EOC

**Fig. 1:**
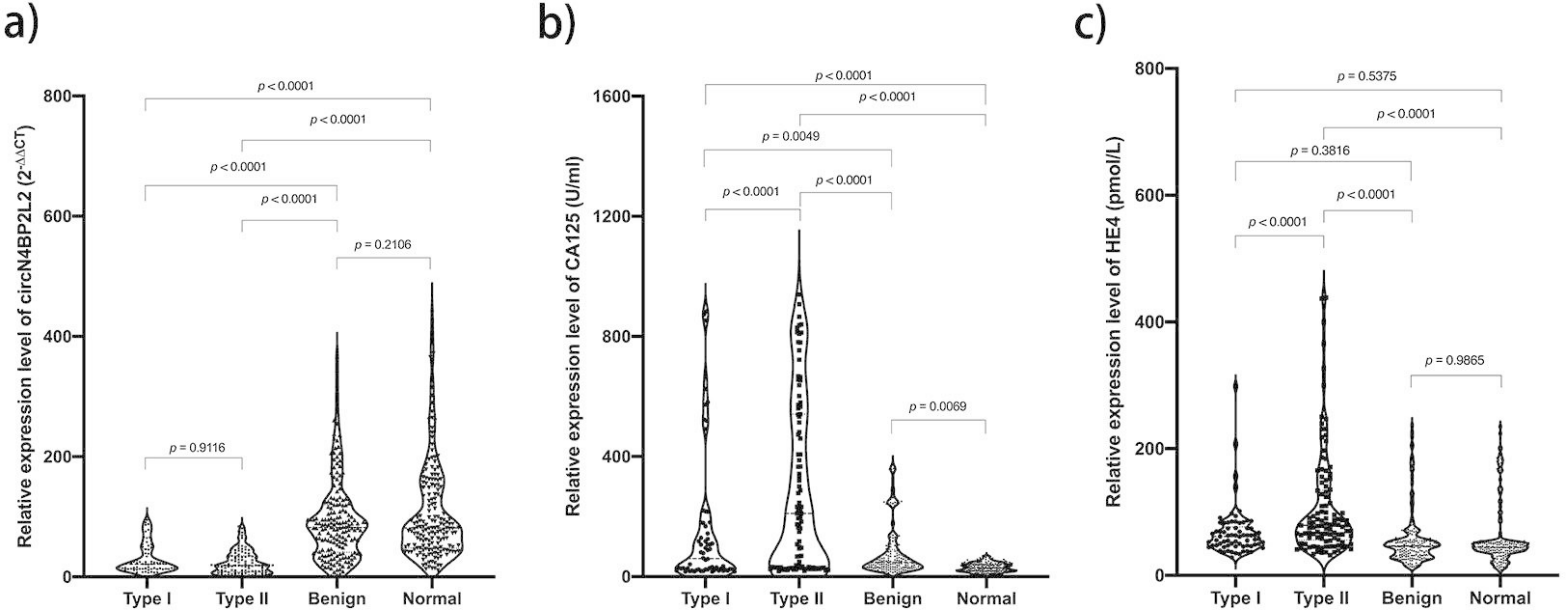
Relative expression level of plasma circN4BP2L2 (a), CA125 (b) and HE4 (c) in type I EOC (n = 70), type II EOC (n = 110), benign ovarian cysts (n = 180) and normal controls (n = 180)

The relative expression level of circN4BP2L2 was significantly lower in both type I and type II EOC, when compared to those in benign and normal cohort (*p* < 0.01). Conversely, the relative expression level of CA125 was significantly higher in both type I and type II EOC, when in comparison with those in benign and normal cohort (*p* < 0.01). However, when comparing with the benign and normal cohorts, the relative expression level of HE4 was higher in type II EOC (*p* < 0.01), but not in type I cohort (*p* > 0.05). Besides, CA125 and HE4 levels were significantly different between type I and type II cohorts (*p* < 0.01), but not circN4BP2L2 (*p* = 0.91). CA125 (*p* < 0.01), but not circN4BP2L2 (*p* = 0.21) or HE4 (*p* = 0.99), was significant when benign cohort was compared to normal cohort (Fig. 1).

### The diagnostic value of circN4BP2L2 in type I EOC

Both circN4BP2L2 and HE4 could separate type I EOC from benign cohort, whereas CA125 could only distinguish late-stage type I EOC from benign cohort. Besides, circN4BP2L2, CA125 and HE4 could all separate type I EOC from normal cohort (Table 3). The median value in Type I EOC for HE4 was 61 pmol/L, and decreased to 46 pmol/L and 44 pmol/L in benign and normal cohorts, respectively. The decrease was even more notable for CA125 where median value of CA125 ranged from 85 U/ mL in type I EOC to 48 U/ mL in benign and 29 U/ mL in normal cohort. The median value in type I EOC for circN4BP2L2 was 21, and increased to 82 in both benign and normal cohort.

**Table 3:** CircN4BP2L2, CA125 and HE4 expression levels according to histology, type and stage; ROC AUC, sensitivity, specificity, and significant difference in type I EOC vs. benign and normal cohorts

Type I EOC cohort		Benign cohort					Normal cohort				
	Median (range)	Median (range)	ROC AUC (95% CI)	Sen	Spe	***P***-value	Median (range)	ROC AUC (95% CI)	Sen	Spe	***P***-value
CircN4BP2L2	21 (7–95)	82 (1–367)	0.84 (0.79–0.89)	69%	87%	< 0.01^*^	82 (11–439)	0.87 (0.82–0.92)	69%	93%	< 0.01^*^
Early-stage ^a^	25 (7–91)		0.79 (0.72–0.85)	90%	56%	< 0.01^*^		0.81 (0.75–0.88)	55%	93%	< 0.01^*^
Late-stage ^a^	15 (11–95)		0.92 (0.86–0.97)	87%	94%	< 0.01^*^		0.95 (0.90–0.99)	87%	98%	< 0.01^*^
CA125	85 (16–5420)	48 (13–373)	0.55 (0.46–0.65)	59%	32%	0.20	29 (4–69)	0.73 (0.65–0.81)	59%	58%	< 0.01^*^
Early-stage ^a^	33 (16–181)		0.40 (0.30–0.51)	48%	32%	0.06		0.64 (0.53–0.75)	48%	58%	< 0.01^*^
Late-stage ^a^	506 (18–5420)		0.75 (0.62–0.88)	73%	32%	< 0.01^*^		0.85 (0.75–0.94)	73%	58%	< 0.01^*^
HE4	61 (28–298)	46 (12–237)	0.70 (0.63–0.76)	54%	76%	< 0.01^*^	44 (7–235)	0.70 (0.63–0.77)	54%	82%	< 0.01^*^
Early-stage ^a^	50 (28–207)		0.62 (0.53–0.70)	38%	76%	0.02^*^		0.63 (0.54–0.71)	38%	82%	0.01^*^
Late-stage ^a^	69 (43–298)		0.80 (0.73–0.87)	77%	76%	< 0.01^*^		0.80 (0.74–0.87)	77%	82%	< 0.01^*^

^a^ According to International Federation of Gynecology and Obstetrics staging

Abbreviations: EOC, epithelial ovarian cancer; ROC, receiver operating characteristic curve; AUC, area under curve; 95% CI, 95% confidence interval; Sen, sensitivity; Spe, specificity

**Fig. 2:**
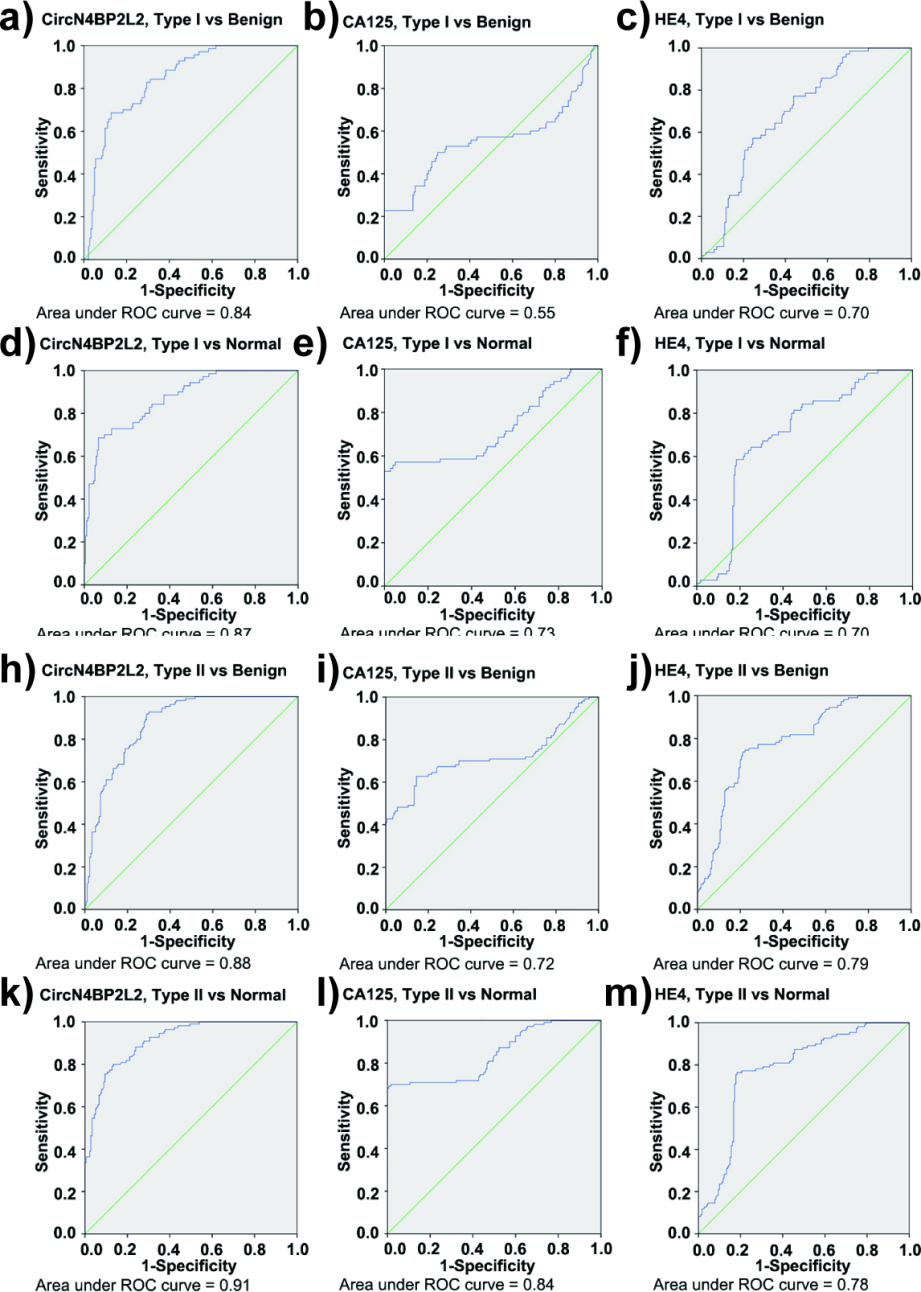
ROC AUC for circN4BP2L2, CA125 and HE4 in type I and type II EOC. It contains ROC AUC for circN4BP2L2 comparing type I EOC with benign (**a**) and normal (**d**) cohorts; ROC AUC for CA125 comparing type I EOC with benign (**b**) and normal (**e**) cohorts; ROC AUC for HE4 comparing type I EOC with benign (**c**) and normal (**f**) cohorts; ROC AUC for circN4BP2L2 comparing type II EOC with benign (**h**) and normal (**k**) cohorts; ROC AUC for CA125 comparing type II EOC with benign (**i**) and normal (**l**) cohorts; and ROC AUC for HE4 comparing type II EOC with benign (**j**) and normal (**m**) cohorts

**Fig. 3:**
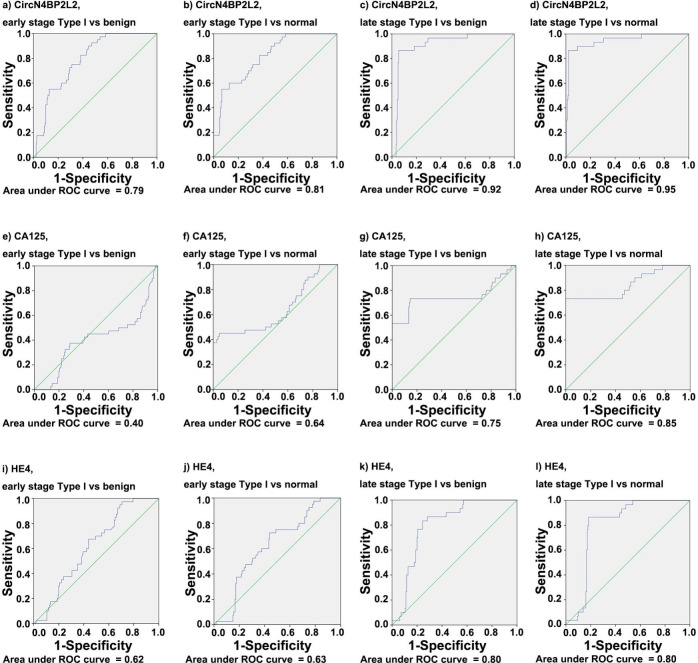
ROC AUC for circN4BP2L2, CA125 and HE4 in type I EOC with regard to tumor stage. It contains ROC AUC for circN4BP2L2 comparing early-stage type I EOC with benign (**a**) and normal (**b**) cohorts; ROC AUC for circN4BP2L2 comparing late-stage type I EOC with benign (**c**) and normal (**d**) cohorts; ROC AUC for CA125 comparing early-stage type I EOC with benign (**e**) and normal (**f**) cohorts; ROC AUC for CA125 comparing late-stage type I EOC with benign (**g**) and normal (**h**) cohorts; ROC AUC for HE4 comparing early-stage type I EOC with benign (**i**) and normal (**j**) cohorts; and ROC AUC for HE4 comparing late-stage type I EOC with benign (**k**) and normal (**l**) cohorts

In discrimination between type I EOC and benign cohorts, the ROC AUC was high for circN4BP2L2 (0.84) (Fig. 2a), followed by HE4 (0.70) (Fig. 2c) and CA125 (0.55) (Fig. 2b). The sensitivity and specificity were higher for circN4BP2L2 (69%, 87%) than those for CA125 (59%, 32%) and HE4 (54%, 76%). When comparing early-stage tumors with benign cohort, the ROC AUC was high for circN4BP2L2 (0.79 circN4BP2L2; 0.62 HE4; 0.40 CA125) (Fig. 3a, i and e), the sensitivity was also high for circN4BP2L2 (90% circN4BP2L2; 48% CA125; 38% HE4), and the specificity was high for HE4 (76% HE4; 56% circN4BP2L2; 32% CA125). When comparing late-stage tumors with benign cohort, the ROC AUC was high for circN4BP2L2 (0.92 circN4BP2L2; 0.80 HE4; 0.75 CA125) (Fig. 3c and k g), the sensitivity was also high for circN4BP2L2 (87% circN4BP2L2; 77% HE4; 73% CA125), and the specificity was also high for circN4BP2L2 (94% circN4BP2L2; 76% HE4; 32% CA125).

When distinguishing type I EOC from normal cohort, the ROC AUC was high for circN4BP2L2 (0.87) (Fig. 2d), followed by CA125 (0.73) (Fig. 2e) and HE4 (0.70) (Fig. 2f). The sensitivity and specificity were higher for circN4BP2L2 (69%, 93%) than those for HE4 (54%, 82%) and CA125 (59%, 58%). In discrimination between early-stage tumors and normal cohort, the ROC AUC was high for circN4BP2L2 (0.81 circN4BP2L2; 0.64 CA125; 0.63 HE4) (Fig. 3b, f and j), the sensitivity was also high for circN4BP2L2 (55% circN4BP2L2; 48% CA125; 38% HE4), and the specificity was also high for circN4BP2L2 (93% circN4BP2L2; 82% HE4; 58% CA125). In discrimination between late-stage tumors and normal cohort, the ROC AUC was high for circN4BP2L2 (0.95 circN4BP2L2; 0.85 CA125; 0.80 HE4) (Fig. 3d h, Fig. 3 L), the sensitivity was also high for circN4BP2L2 (87% circN4BP2L2; 77% HE4; 73% CA125), and the specificity was also high for circN4BP2L2 (98% circN4BP2L2; 82% HE4; 58% CA125).

### The diagnostic value of circN4BP2L2 in type II EOC

When separating type II EOC from benign or normal cohort, statistical significance was achieved between all groups, except for CA125 in distinguishing early-stage tumors from benign cohort (Table 4). The median value for circN4BP2L2 in type II EOC was 19, and increased to 82 in both benign and normal cohort. The median value for HE4 in type II EOC was 79 pmol/L, and decreased to 46 pmol/L and 44 pmol/L in benign and normal cohorts, respectively. The decrease was even more notable for CA125 where median value of CA125 ranged from 233 U/ mL in type II EOC cohort to 48 U/ mL in benign cohort and 29 U/ mL in normal cohort.

**Table 4:** CircN4BP2L2, CA125 and HE4 expression levels according to histology, type and stage; ROC AUC, sensitivity, specificity, and significant difference in type II EOC vs. benign and normal cohorts

Type II EOC cohort		Benign cohort					Normal cohort				
	Median (range)	Median (range)	ROC AUC (95% CI)	Sen	Spe	***P***-value	Median (range)	ROC AUC (95% CI)	Sen	Spe	*P*-value
CircN4BP2L2	19 (1–84)	82 (1–367)	0.88 (0.84–0.91)	93%	71%	< 0.01^*^	82 (11–439)	0.91 (0.88–0.94)	80%	87%	< 0.01^*^
Early-stage ^a^	24 (1–74)		0.85 (0.79–0.92)	88%	67%	< 0.01^*^		0.89 (0.83–0.95)	83%	77%	< 0.01^*^
Late-stage ^a^	19 (2–84)		0.88 (0.84–0.92)	94%	69%	< 0.01^*^		0.92 (0.89–0.95)	83%	87%	< 0.01^*^
CA125	233 (18–5801)	48 (13–373)	0.72 (0.65–0.79)	72%	32%	< 0.01^*^	29 (4–69)	0.84 (0.79–0.89)	72%	58%	< 0.01^*^
Early-stage ^a^	30 (18–560)		0.46 (0.31–0.60)	46%	32%	0.49		0.68 (0.55–0.80)	46%	58%	< 0.01^*^
Late-stage ^a^	398 (22–5801)		0.79 (0.72–0.86)	79%	32%	< 0.01^*^		0.89 (0.84–0.94)	79%	58%	< 0.01^*^
HE4	79 (32–438)	46 (12–237)	0.79 (0.73–0.84)	75%	76%	< 0.01^*^	44 (7–235)	0.78 (073–0.84)	75%	82%	< 0.01^*^
Early-stage ^a^	88 (34–231)		0.77 (0.68–0.87)	71%	76%	< 0.01^*^		0.75 (0.65–0.85)	71%	82%	< 0.01^*^
Late-stage ^a^	78 (32–438)		0.79 (0.74–0.85)	77%	76%	< 0.01^*^		0.79 (0.73–0.85)	77%	82%	< 0.01^*^

^a^ According to International Federation of Gynecology and Obstetrics staging

Abbreviations: EOC, epithelial ovarian cancer; ROC, receiver operating characteristic curve; AUC, area under curve; 95% CI, 95% confidence interval; Sen, sensitivity; Spe, specificity

**Fig. 4:**
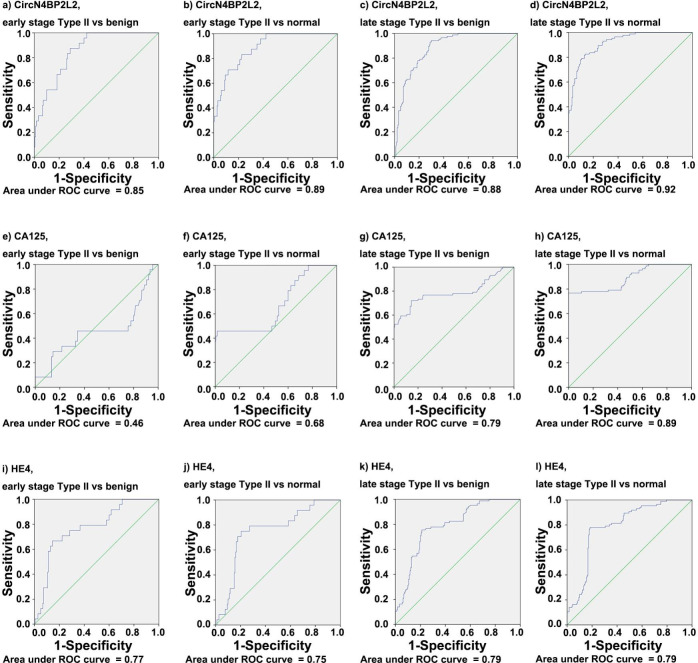
ROC AUC for circN4BP2L2, CA125 and HE4 in type II EOC with regard to tumor stage. It contains ROC AUC for circN4BP2L2 comparing early-stage type II EOC with benign (**a**) and normal (**b**) cohorts; ROC AUC for circN4BP2L2 comparing late-stage type II EOC with benign (**c**) and normal (**d**) cohorts; ROC AUC for CA125 comparing early-stage type II EOC with benign (**e**) and normal (**f**) cohorts; ROC AUC for CA125 comparing late-stage type II EOC with benign (**g**) and normal (**h**) cohorts; ROC AUC for HE4 comparing early-stage type II EOC with benign (**i**) and normal (**j**) cohorts; and ROC AUC for HE4 comparing late-stage type II EOC with benign (**k**) and normal (**l**) cohorts

In discrimination between type II EOC and benign cohorts, the ROC AUC was high for circN4BP2L2 (0.88) (Fig. 2 h), followed by HE4 (0.79) (Fig. 2j) and CA125 (0.72) (Fig. 2i). The sensitivity and specificity were higher for circN4BP2L2 (93%, 71%) than those for HE4 (75%, 76%) and CA125 (72%, 32%). When comparing early-stage tumors with benign cohort, the ROC AUC was high for circN4BP2L2 (0.85 circN4BP2L2; 0.77 HE4; 0.46 CA125) (Fig. 4a, i and e), the sensitivity was also high for circN4BP2L2 (88% circN4BP2L2; 71% HE4; 46% CA125), and the specificity was high for HE4 (76% HE4; 67% circN4BP2L2; 32% CA125). When comparing late-stage tumors with benign cohort, the ROC AUC was high for circN4BP2L2 (0.88 circN4BP2L2; 0.79 CA125; 0.79 HE4) (Fig. 4c g, Fig. 4k), the sensitivity was also high for circN4BP2L2 (94% circN4BP2L2; 79% CA125; 77% HE4), and the specificity was high for HE4 (76% HE4; 69% circN4BP2L2; 32% CA125).

When distinguishing type II EOC from normal cohort, the ROC AUC was high for circN4BP2L2 (0.91) (Fig. 2k), followed by CA125 (0.84) (Fig. 2 L) and HE4 (0.78) (Fig. 2 m). The sensitivity and specificity were higher for circN4BP2L2 (80%, 87%) than those for HE4 (75%, 82%) and CA125 (72%, 58%). In discrimination between early-stage tumors and normal cohort, the ROC AUC was high for circN4BP2L2 (0.89 circN4BP2L2; 0.75 HE4; 0.68 CA125) (Fig. 4b, j and f), the sensitivity was also high for circN4BP2L2 (83% circN4BP2L2; 71% HE4; 46% CA125), and the specificity was high for HE4 (82% HE4; 77% circN4BP2L2; 58% CA125). In discrimination between late-stage tumors and normal cohort, the ROC AUC was high for circN4BP2L2 (0.92 circN4BP2L2; 0.89 CA125; 0.79 HE4) (Fig. 4d h, Fig. 4 L), the sensitivity was also high for circN4BP2L2 (83% circN4BP2L2; 79% CA125; 77% HE4), and the specificity was also high for circN4BP2L2 (87% circN4BP2L2; 82% HE4; 58% CA125).

## Discussion

Traditionally, EOC is classified based on histologic features [6]. Recently, it has been proposed that EOC should be classified into slow-growing type I and more aggressive type II tumors according to both histologic features and molecular genetics [8]. Previous studies reported that CA125 and HE4 were highly representative diagnostic biomarkers for type II EOC; however, both markers showed limited diagnostic ability in all type I tumors and early-stage type II malignancies [10, 29]. In this study, we aimed to assess the diagnostic value of circN4BP2L2 in type I and type II EOC. The blood samples of a cohort of 540 women that were prospectively and consecutively collected were used for analysis. Our data suggested that circN4BP2L2 had a good diagnostic ability in both type I and type II EOC. In comparison to the benign or normal cohort, circN4BP2L2 resulted in good diagnostic power with ROC AUC 0.84 and 0.87 in type I, and 0.88 and 0.91 in type II tumors, and impressively high AUC 0.95 for late-stage type I EOC in comparison to the normal cohort. Notably, circN4BP2L2 also had good diagnostic performance in early-stage tumors in both type I and type II. To our knowledge, circN4BP2L2 has not earlier been evaluated by dividing EOC into type I and type II tumors.

Consistent with our results, the diagnostic value of circRNAs has been previously proved in various malignancies. Plasma circFARSA is significantly upregulated in non-small cell lung cancer and might serve as a promising noninvasive diagnostic biomarker for this malignancy [30]. Circ_0001785 is significantly dysregulated in breast cancer and was closely associated with tumor stage and distant metastasis, plasma circ_0001785 had better diagnostic performance than CA15-3 and CEA in breast cancer patients [31]. Circ_0000190, which was previously discovered to be down-regulated in gastric cancer tissues by microarray screening, had better AUC, sensitivity and specificity than commonly used diagnostic biomarkers such as CA19-9 and CEA [32]. The expression level of circ_0003998 was significantly increased in patients with hepatocellular carcinoma, and plasma circ_0003998 had a good diagnostic accuracy in distinguishing cancer patients from hepatitis B patients (AUC = 0.83) and healthy controls (AUC = 0.89) [33]. Similar results have also been achieved in colorectal cancer [34], esophageal squamous cell carcinoma [35] and prostate cancer [36].

The ultimate goal to improve EOC prognosis is to detect early-stage cases regardless of tumor subtype. In our dataset, early stage EOC comprised 35% of the malignant cohort, which is in accordance with previous studies [37, 38]. While CA125 and HE4 showed limited diagnostic value for recognizing early-stage tumors in both type I and type II EOC, circN4BP2L2 had a good diagnostic ability for early-stage cases. In comparison to the benign or normal cohort, circN4BP2L2 exhibited good diagnostic performance with ROC AUC 0.79 and 0.81 in early-stage type I, and 0.85 and 0.89 in early-stage type II EOC. Conventional techniques for detecting ovarian tumors include gynecological bimanual palpation, transvaginal ultrasound and computed tomography scanning [39]. It has been proposed that type I tumors, which are more often localized in the pelvis and more generally of a larger size, are more easily to be detected at an earlier stage with conventional techniques than type II tumors, which are more often of invisible early lesions [40, 41]. Notably, our data revealed that circN4BP2L2 had even better diagnostic performance in distinguishing early-stage type II EOC than that in distinguishing early-stage type I EOC. These results suggested that circN4BP2L2 might serve as an adjunct to conventional techniques for detecting early stage EOC cases. Further large-scale clinical trials are warranted to evaluate the practicability of circN4BP2L2 for clinical application.

By assessing the association between the expression level of circN4BP2L2 and clinicopathological features of EOC, we found that low expression of circN4BP2L2 was correlated to advanced stage, poor differentiation and LNM. In stratified analysis, the data revealed that decreased circN4BP2L2 was respectively associated with advanced stage in Type I tumor and LNM in type II tumor. Additionally, our in vitro experiments revealed that decreased circN4BP2L2 might improve cell metastasis and invasion in EOC. Both clinical analyses and fundamental researches suggested that circN4BP2L2 participated in EOC development via regulating tumor cell metastasis. These results are in accordance with other published articles, where they concluded that circRNAs might promote EOC progression by regulating cell proliferation, apoptosis or migration [23–26]. With regard to the function mechanism of circRNAs in ovarian cancer, the most studied one was for circRNAs acting as microRNA sponges. It has been reported that circUBAP2 [42], circPLEKHM3 [43], and circMYLK [44] could respectively promote ovarian cancer development via regulating miR-382-5p, miR-9 and miR-652. Besides, circRNAs could participate in ovarian cancer development by targeting and regulating RNA-binding proteins. Liu et al. [45] reported that circ_0005276 was significantly upregulated in EOC and could aggravate the development of EOC by binding ADAM9. Chen et al. [46] discovered that circNOLC1 could promote EOC tumorigenesis and progression by binding ESRP1 and modulating the expression of CDK1 and RhoA. Furthermore, Yan et al. [47] reported that circITCH could inhibit the proliferation of ovarian cancer via downregulating lncRNA HULC. Despite the abovementioned mechanisms, the exact function mechanism of circN4BP2L2 is still unknown. Further laboratory experiments are needed to clarify how circN4BP2L2 functions in EOC progression.

Despite its rarity, type I EOC is a group of individually different tumors of clinical importance. Advanced mucinous and clear cell carcinomas are quite aggressive and have even higher mortality than type II EOC. Neither CA125 nor HE4 could effectively detect these type I tumors. Accordingly, our data revealed that the expression level of CA125 and HE4 in type II tumors were significantly higher than those in type I tumors, and both markers failed to separate type I tumors from benign or normal cohort. Notably, we found that the expression level of circN4BP2L2 in type I EOC was similar to that in type II EOC. When compared to benign or normal cohort, circN4BP2L2 was significantly decreased in both type I and type II tumors. The diagnostic performance of circN4BP2L2 was equally well in type I and type II. These results suggested that circN4BP2L2 might serve as an early biomarker for diagnosing all histology subtypes, including mucinous and clear cell tumors. However, due to limited number of type I cases, we were unable to conduct stratified analysis based on different histology. Further larger-scale clinical trials are needed to investigate the expression level of circN4BP2L2 in different histological subtype.

CA125 and HE4 are two FDA-approved EOC biomarkers. Instead of being used for disease detection, they are more usually applied for efficacy evaluating and recurrence monitoring. Except for relatively low sensitivity in early-stage cases, CA125 also had poor specificity in diagnosing EOC. It has been reported that, apart from EOC, elevated CA125 could also be tested in non-EOC gynecological malignancies such as endometrial cancer and cervical cancer, non-gynecological malignancies such as lung cancer and breast cancer, benign gynecological diseases such as endometriosis, cardiopulmonary diseases such as myocardial infarction and chronic obstructive pulmonary disease, pregnant women, and even healthy women [48, 49]. Particularly, CA125 could elevate in approximately half of the patients with endometriosis [50]. Therefore, the value of CA125 in distinguishing EOC from benign ovarian cysts is often limited. In our series, more than half of the patients (104/180, 58%) in benign cohort had endometriosis, which might partly explain the poor diagnostic value of CA125 in separating EOC from benign cohort. While HE4 has relatively high specificity in diagnosing EOC, its sensitivity is also unsatisfactory [16]. Besides, controversies arise regarding whether the performance of HE4 could be affected by menopausal status. Some researchers reported that HE4 had better diagnostic value in post-menopausal ovarian cancer patients [51, 52], while others believed that HE4 performed equally well in both pre- and post-menopausal ovarian cancer patients [53, 54]. By and large, both markers showed limited value in diagnosing EOC.

This study has two main limitations. On one hand, the sample size was relatively small and we were unable to conduct stratified analysis based on tumor histology. On the other hand, the evaluation of circN4BP2L2 as a true diagnostic biomarker was limited in our cohort of patients with suspicious malignancies or benign ovarian cysts, formal study using a screening cohort is needed in the future.

## Conclusion

Our results demonstrated that circN4BP2L2 might serve as a promising diagnostic biomarker for both type I and type II EOC. The diagnostic safety for circN4BP2L2 in early-stage type I and type II EOC is also acceptable. Further large-scale well-designed studies are warranted to investigate whether circN4BP2L2 is specific for all histologic subgroups.

## Electronic supplementary material

Below is the link to the electronic supplementary material.

Supplementary Material 1

## Data Availability

The datasets used and/or analyzed during the current study are available from the corresponding author on reasonable request.

## Abbreviations

EOC: Epithelial ovarian cancer
RT-qPCR: Reverse transcription-quantitative polymerase chain reaction
CA125: Cancer antigen 125
HE4: Human epididymis protein 4
ELISA: Enzyme-linked immunosorbent assay
ROC: Receiver operating curve
AUC: The area under the curve
LNM: Lymph node metastasis
OC: ovarian cancer
G: grade
FDA: the US Food and Drug Administration
CircRNAs: Circular RNAs
CAMS: Chinese Academy of Medical Sciences
FIGO: The International Federation of Gynecology and Obstetrics
sen: Sensitivity
spe: Specificity
CA15-3: Cancer antigen 15 − 3
CEA: Carcinoembryonic antigen
CA19-9: Cancer antigen 19 − 9
