# Supplementary material for: Diagnostic value of circN4BP2L2 in type I and type II epithelial ovarian cancer
Source: BMC Cancer. 2022 Nov 24;22:1210. doi: 10.1186/s12885-022-10138-w (PMC9694909; doi:10.1186/s12885-022-10138-w)
Supplement: Supplementary file 1 — Supplementary Material 1 [file 12885_2022_10138_MOESM1_ESM.docx]

**Diagnostic value of circN4BP2L2 in type I and type II epithelial ovarian cancer**

Li Ning ^a^, Jinghe Lang ^b^, Bo Long ^b,*^, Lingying Wu ^a,*^

^a^ Department of gynecologic oncology, National Cancer Center/National Clinical Research Center for Cancer/Cancer Hospital, Chinese Academy of Medical Sciences and Peking Union Medical College, Beijing, 100021, China.

^b^ Peking Union Medical College Hospital, Chinese Academy of Medical Sciences and Peking Union Medical College, Beijing, 100730, China.

^*^ Corresponding author. E-mail address: longbopumch@126.com (Bo Long), a12124537@126.com (Lingying Wu).

**Supplementary Table S1.** The cut-off value of circN4BP2L2 calculated by Youden index in each comparison group.

| Comparison group | Cut-off value of circN4BP2L2 |
| --- | --- |
| Type I EOC vs benign | 27.98 |
| Type I EOC vs normal | 28.035 |
| Type II EOC vs benign | 53.705 |
| Type II EOC vs normal | 39.845 |
| Type I early stage EOC vs benign | 78.455 |
| Type I early stage EOC vs normal | 28.035 |
| Type I late stage EOC vs benign | 16.47 |
| Type I late stage EOC vs normal | 16.335 |
| Type II early stage EOC vs benign | 53.705 |
| Type II early stage EOC vs normal | 45.845 |
| Type II late stage EOC vs benign | 56.565 |
| Type II late stage EOC vs normal | 39.845 |

Abbreviation: EOC, epithelial ovarian cancer.
